# Recent Developments in Nanomedicine for Pediatric Cancer

**DOI:** 10.3390/jcm10071437

**Published:** 2021-04-01

**Authors:** Shicheng Yang, Mia Wallach, Apurva Krishna, Raushan Kurmasheva, Srinivas Sridhar

**Affiliations:** 1 Department of Chemical Engineering, Northeastern University, Boston, MA 02115, USA; yang.shic@northeastern.edu; 2 School of Business, Northeastern University, Boston, MA 02115, USA; wallach.m@northeastern.edu; 3 Department of Physics, Northeastern University, Boston, MA 02115, USA; krishna.ap@northeastern.edu; 4 Department of Molecular Medicine, The University of Texas Health at San Antonio, San Antonio, TX 78229, USA; 5 Greehey Children’s Cancer Research Institute, San Antonio, TX 78229, USA; 6 Division of Radiation Oncology, Harvard Medical School, Boston, MA 02115, USA

**Keywords:** pediatric cancer, nanoparticles, drug delivery system, liposome, leukemia, lymphoma, osteosarcoma, Ewing sarcoma, glioma, blood–brain barrier

## Abstract

Cancer is the second biggest cause of death in children in the US. With the development of chemotherapy, there has been a substantial increase in the overall survival rate in the last 30 years. However, the overall mortality rate in children with cancer remains 25%, and many survivors experience a decline in overall quality of life and long-term adverse effects caused by treatments. Although cancer cells share common characteristics, pediatric cancers are different from adult cancers in their prevalence, mutation load, and drug response. Therefore, there is an urgent unmet need to develop therapeutic approaches specifically designed for children with cancer. Nanotechnology can potentially overcome the deficiencies of conventional methods of administering chemotherapy and ultimately improve clinical outcomes. The nanoparticle-based drug delivery systems can decrease the toxicity of therapy, provide a sustained or controlled drug release, improve the pharmacokinetic properties of loading contents, and achieve a targeted drug delivery with achievable modifications. Furthermore, therapeutic approaches based on combining nanoformulated drugs with novel immunotherapeutic agents are emerging. In this review, we discussed the recently developed nanotechnology-based strategies for treating blood and solid pediatric cancers.

## 1. Introduction to Pediatric Cancer

In the United States, pediatric cancer is the second biggest cause of death for children under the age of 14, just exceeded by accidents [1]; and it is a leading cause of death for children and adolescents worldwide, particularly in high-income countries [2]. Since the mid-1970s, pediatric cancer has been a salient research topic, and new research on the subject is frequently emerging. There has been a substantial decline in the mortality rates for various cancers over the last 30 years among children under the age of 19 [3,4]. As a result, the 5-year survival rate increased from 58% (in the mid-1970s) to 84% by 2021. However, despite recent progress, the 5-year death rate in children with cancer remains high, and many survivors experience long-term adverse effects that worsen their quality of life [1,5].

### 1.1. Traditional and Modern Chemotherapy

Cytotoxic chemotherapy utilizing DNA alkylating agents and antimetabolites, has been the most widely used cancer treatment in the past 50 years. Current chemotherapies produce a host of unintended effects that can pose lifelong implications for survivors, such as severe sequelae and decreased quality of life. Common side effects of current chemotherapies include fatigue, nausea, diarrhea, mouth sores, hair loss, and anemia. These apoptosis-inducing therapies cannot differentiate rapidly dividing normal cells from cancerous cells causing systemic toxicity. Furthermore, chemotherapy is typically administered daily through oral or intravenous injection, which constrains patients to daily medical appointments or inpatient care and further reduces their quality of life.

A deeper understanding of cancer biology in recent decades has accelerated the design of molecules that target and inhibit proteins (such as v-raf murine sarcoma viral oncogene homolog B1 or BRAF) and pathways (like angiogenesis, blocking DNA repair or inducing DNA damage) that are crucial for tumor growth or contribute to cancer cell proliferation [6]. In the last 30 years, the U.S. Food and Drug Administration (FDA) has approved over 30 small molecules for cancer treatment. Most of these compounds are inhibitors with molecular weights of less than 500 Daltons that aim to slow or stop the cell cycle and lead to the eventual death of cancer cells. Inhibitors induce apoptosis by blocking key receptors or enzymes, interfering with downstream intracellular signaling molecules, introducing genetic damage, or preventing DNA repair [7]. These chemotherapies can often shrink or delay the growth of solid tumors, which allows patients to live longer with a better quality of life. Additionally, chemotherapy can reduce the possibility of cancer recurrence after tumor reduction surgery. 

### 1.2. Critical Differences between Pediatric and Adult Cancers

Cancer is an extremely heterogeneous disease with complex and tissue-specific impairments from genetic, epigenetic, and environmental factors. Understanding the critical differences between pediatric and adult cancers is integral for investigating the underlying pathophysiologic and molecular mechanisms for developing new diagnostic/therapeutic approaches [8].

First, types of cancers are different between children and adults. Childhood cancers are not triggered by lifestyle as in adults, and very few are inherited from parents. Although the risk of developing most types of cancer significantly increases with age, there are several exceptions. For example, while brain and bone cancers are rare in adults, they have much higher rates in children [9,10]. Similarly, leukemia is also more prevalent in children; it accounts for 28% of all pediatric cancers, but only 3% to 4% of cancers among all age groups [10,11].

Second, even though cancers in children tend to respond better to therapy, children and adults differ in genitourinary pH and transit, intestinal motility and conjugation, and transport of bile salts. All of these factors affect the metabolism of chemotherapeutic drugs. Pediatric tissues and organs are immature, and because children are rapidly developing, they have higher metabolic rates. Treatment dosages that are safe to administer to adults are often severely toxic to a child’s developing organs, which absorb, distribute, and eliminate substances more rapidly than the same type of organ in an adult. 

Finally, genetic stability in children and adults is also different, which affects mutation rates in cancer [12]. In a study of pan-cancer genome and transcriptome analyses of all reported driver genes in pediatric cancer, only 45% matched those found in adult cancers [13,14]. Children have ‘quieter’ genomes, and many pediatric cancers, such as Ewing sarcoma, alveolar rhabdomyosarcoma, synovial sarcoma, and acute lymphocytic leukemia (ALL), are driven by fusion oncogenes resulting from chromosomal translocations [15,16,17,18]. Compared to children, adult genomes have a much higher mutational rate, which provides unique molecular targets for treatment strategies. The lack of mutation targets makes the development of targeting treatments for pediatric cancer more challenging. Thus, types of mutations in pediatric cancer can differ from those in adults, leading to a different integration of chemotherapies with drug targets, and in turn making the strategy of targeting specific mutations less effective for children. 

For the above reasons, chemotherapy doses in children cannot be simplified by the direct adjustment to the body weight/surface, as they often are in adults. Therefore, there is an urgent unmet need to develop therapeutic methods specifically designed for children with cancer, which may provide clinicians with more powerful weapons to treat them.

Significant differences between the participation of children and adults in clinical trials using nanocarriers are found through the search engine ClinicalTrial.gov. There are 1523 studies for liposome as treatments for cancer in adults, while there are just 364 studies for all age groups (including children). If we search for “pediatric” and “liposome”, 33 results were obtained, and only 11 of them are for pediatric cancer, with 10 studies being specially designed for children (excluding adults), as shown in Table 1 (data are collected by 11 March 2021).

### 1.3. Challenges in Pediatric Cancer Treatment

While clinical trials in children are more complicated due to scientific, ethical, and technical factors that have stunted research progress in recent years, the lack of studies about drug disposition in pediatric patients poses a key difficulty for clinical applications. The extrapolation of adult dosing is often used to determine the dosage for pediatric patients. However, the pharmacokinetic and pharmacodynamic properties of roughly 70% of drugs prescribed to pediatric patients have not been appropriately studied in children, necessitating the development of new approaches for determining the dosage for pediatric patients [19]. 

Over the past several decades, astounding progress has been made in the development of chemotherapy and the emergence of novel treatments for the adults, such as immunotherapy and combination therapy. Nevertheless, conventional anti-cancer treatments can have late side effects or cause severe long-term health problems that are not well studied once former pediatric cancer patients mature into adults [8]. Consequently, there is a pressing need to develop targeted therapies or drug carriers that can deliver therapeutic agents with higher efficiency to lower the dosage needed and minimize side effects. 

One of the significant recent developments aimed to advance pediatric research into clinical development is the Research to Accelerate Cures and Equity for Children Act (RACE for Children Act) [20,21,22] and the public–private partnership [23,24,25], which aims to necessitate having sufficient pediatric models with the appropriate genetic alterations. The RACE Act requires the FDA to develop a list of the molecular targets of known and new drugs, and pediatric agents substantially relevant to cancer growth and progression will require pediatric trials. This expectation applies both to drugs or biologics developed by pharmaceutical companies and by academic institutions.

## 2. Nanomedicine

### 2.1. Introduction to Nanomedicine

Nanotechnology is an emerging interdisciplinary strategy in cancer diagnosis and treatment. Nanotechnology has rapidly developed over the past ten years and has been intensively applied in fields of engineering, medicine, biology, and chemistry. For example, nanoparticles are widely used as a vehicle for the delivery of drugs as well as vaccines, or directly act as therapeutic agents for certain diseases [26,27]. Moreover, there are novel features that are being explored by researchers for the continuous monitoring of drug distribution, such as fighting against viruses like COVID-19 and implantable nanosensors [28,29,30]. In addition, nanoparticles have been considered as a novel strategy not only in therapeutics but also in the diagnosis of cancer research [31,32]. 

### 2.2. Nanoparticles as a Delivery Method

**Figure 1:**
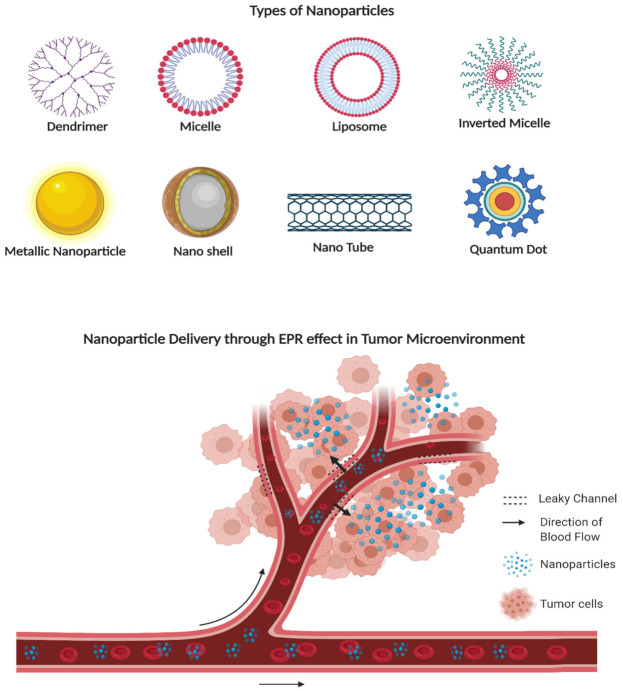
Types of nanoparticles and the enhanced permeability and retention (EPR) effect: top, nanoparticles that are commonly used in anti-cancer treatment; bottom, tumor vasculature and dysfunctional lymphatic drainage allows the accumulation of nanoparticles in the tumor cell environment. (Created with BioRender.com at 6 March 2021).

Nanoparticles, defined as particles ranging from 1 to 1000 nm, have many unique properties compared to larger particles, and have been widely studied for their potential as anti-cancer drug delivery platforms (Figure 1, top). Nanoparticles can deliver drugs selectively to tumors, and the modification of nanoparticle surfaces allows loaded drug molecules to prevent immune system recognition and elimination by the body. The most often used strategy is fine-tuning the nanodelivery system, in which specially designed nanoparticles are loaded with small drug molecules such as chemotherapeutic agents or inhibitors and directly targeted to the tumor site to block metabolism or knock down protein expression. Nanoparticles can be fabricated with various materials to further increase their encapsulation capacity and modify their surface properties to functionalize the target. Thus, nanoparticles could improve the solubility of hydrophobic drugs and prolong drug circulation time in the bloodstream, which would allow lower effective therapeutic doses and therefore fewer side effects. The additional properties of nanoparticles, such as surface charge and size and particle shape, can induce accumulation in the organs. Nanoparticles with neutral or slightly negative charges exhibit the prolonged circulating half-lives. Highly anionic particles tend to evade clearance from the circulatory system better than highly cationic particles [33]. A large amount of in vitro research has been conducted to understand the optimal nanoparticle size in vitro, and how this might translate to in vivo studies and accumulation in specific organs. In general, nanoparticles between 30 and 60 nm have shown increased cellular uptake, due to their ability to both bind to receptors and effectively induce the membrane wrapping process [34]. There is evidence that large and small nanoparticles are effectively cleared from the circulatory system and have less chance of reaching targeted organs. Large spherical nanoparticles above 200 nm in size have been shown to accumulate in the liver and spleen, while nanoparticles less than 5 nm in size are filtered out by the kidneys [34]. Thus, nanoparticles in the diameter of 10–100 nm are considered suitable for drug delivery leveraging enhanced permeability and retention (EPR) effect in tumors [35,36]. The core of a nanoparticle such as a liposome or micelle can be loaded with a single or combination of therapeutics based on the nature of release, water affinity, and degradation rate. Furthermore, to prolong the circulation time and evasion from the mononuclear phagocyte system (MPS), the nanoparticle surface can be coated with polyethylene glycol (PEG) molecules. Several surface modifications can be performed to make nanoparticles suitable for active or passive delivery. Many such methods are described throughout this review. These known characteristics of nanoparticle absorption continue to contribute to the investigation of passive targeting with new nanoparticle materials and characteristics.

Although the anti-tumor efficacy of traditional chemotherapy drugs, such as anthracyclines or DNA alkylating agents, is well proven, the risk of toxicity in pediatric patients creates clinical limitations. Many promising drugs, such as the cyclin-dependent kinase 12 (CDK12) inhibitor dinaciclib, have limited clinical application because of their short half-life and high toxicity [37]. In addition, some orally administered poly (ADP-ribose) polymerase (PARP) inhibitors, such as talazoparib, must pass through the portal vein, where part of the molecules is degraded before entering the main bloodstream, resulting in lower bioavailability (known as the first pass effect). However, if drugs are encapsulated in specially designed nanocarriers, smaller dosages are needed to generate the same therapeutic effect, which lowers toxicity and accomplishes a more stable blood concentration [38]. 

Tumors can induce the growth of blood vessels to supply their cells with necessary nutrients and oxygen supply, resulting in highly disorganized and abnormal vascular networks containing poorly aligned, defective endothelial cells with wide fenestrations. These tissues lack a smooth muscle layer and adequate lymphatic drainage and have innervation with a broader lumen and impaired functional receptors. Such features improve permeability and increase the accumulation of some nanoparticles, such as liposomes (EPR effect) (Figure 1, bottom) and is considered an advantage for nanocarriers [39]. With more drug molecules retained in the target tissue, a lower systemically administered dose can be given without compromising therapeutic efficacy. Nanoparticle formulations have far superior pharmacokinetics compared with the current chemotherapy delivery methods. Furthermore, specific antibodies can be conjugated onto the surfaces of nanoparticles; in this way, nanoparticles can recognize and selectively accumulate designated cancer cells and deliver the loaded therapeutic molecules with increased accuracy. 

Cancer cells have different metabolic activity and active signaling pathways than normal cells, which leads to a significant divergence in surface receptor expression [26,40,41]. Monotherapies can achieve higher drug levels in tumors by conjugating specially designed antibodies on the surface of nanocarriers targeting tumor cells. This enhances therapeutic efficacy and the reduces side effects compared to the same dosage administered conventionally [40,41,42].

A variety of types of nanoparticles developed for drug delivery or as diagnostic agents are described below.

#### 2.2.1. Metallic Nanoparticles

Metallic nanoparticles range from 1 to 100 nm in size and are frequently used as drug carriers and bioimaging agents. They are useful carriers based on their physicochemical properties, high stability, high reactivity, and photothermic and plasmonic properties [43]. Gold nanoparticles have a large surface-to-volume ratio, and their surface chemistries allow customization to optimize charge, hydrophilicity, and functionality [44]. 

#### 2.2.2. Dendrimers, Micelles, and Liposomes

Polymeric micelles, dendrimers, and liposomes are widely used nanocarriers of hydrophobic drugs used to enhance aqueous solubility and prolong the half-life of chemotherapeutic agents in circulation. These nanoparticles are usually 10–100 nm in size and consist of a hydrophilic polyethylene glycol (PEG) outer shell and a hydrophilic core [45,46]. They are self-assembling in water at a particular concentration and often used for passive targeting as drug carriers. This strategy takes advantage of the EPR effect to deliver increased payloads specifically to tumor sites. 

#### 2.2.3. Iron Oxide Nanoparticles

Superparamagnetic iron oxide nanoparticles have been thoroughly studied as inorganic nanocarrier systems for drug delivery [47,48,49]. Iron oxide nanoparticles are uniquely advantageous as they are non-toxic, biodegradable, biocompatible, and efficiently cleared from the body through the iron metabolism pathway. Iron oxide nanoparticles between 10 and 100 nm are optimal, as particles of this size have shown reduced liver and kidney uptake [50]. Their magnetic behavior also allows them to serve as both contrast agents in MRI imaging for diagnostic purposes and be guided to targeted therapeutic sites by external magnetic fields [51]. As iron oxide nanoparticles are hydrophobic and negatively charged, they are recognized by the phagocytic system and are therefore cleared from the body. Uncoated iron oxide nanoparticles generate dose-dependent cytotoxicity in microbial and murine models [52]. 

#### 2.2.4. Nanotubes 

Carbon nanotubes can be functionalized with bioactive peptides, proteins, nucleic acids, and drugs. When employed in this fashion, they are not immunogenic and display low toxicity in drug delivery. Carbon nanotubes have a high propensity for crossing cellular membranes, resulting in high levels of cellular uptake [53]. Nanotubes between 20 and 30 nm in diameter and 700–1100 nm long are most desirable for cancer cell eradication [53,54].

#### 2.2.5. Quantum Dots

Quantum dots range from 2 to 10 nm and are nanometric semiconductors with distinctive optical properties, including high quantum yield, size-tunable light emission, and good chemical and photo-stability. They are used for fluorescent imaging with increased transmission of visible light through biological tissue for diagnostic purposes, drug delivery, and optical agents in sensor systems of biomarkers. Some quantum dots that contain heavy metals such as cadmium and mercury exhibit high levels of toxicity; this can be reduced by functionalizing the surface of quantum dots with biocompatible molecules [55]. 

Thus, nanoformulations can potentially overcome the deficiencies of conventional methods of administering chemotherapy and improve clinical results. Accordingly, the key advantages of the nanoformulations are:

1. More precise dosing in preclinical studies compared to free drugs;
2. Higher dose with less toxicity;
3. Improved pharmacokinetic properties of drugs;
4. More selective, antibody-targeted drug delivery for cancers with specific surface protein expression.

## 3. Blood Cancers

### 3.1. Leukemia

Leukemias account for about 28% of all pediatric malignancies, and are the most common cancers in children [10]. The disease is characterized by abnormal white blood cells that originated from tissues that produce blood cells, such as bone marrow.

Lipid-based nanoparticles have been tested to treat leukemia. One study reported the use of lipid-based solid nanoparticles to deliver mitoxantrone and a P-glycoprotein (P-gp) inhibitor β-element to overcome multidrug resistance [56]. P-gp overexpression is believed to block the mechanism of multidrug resistance. Thus, the co-delivery of the P-gp and mitoxantrone inhibitor can synergistically affect inhibition and maximize treatment effects. This nanocarrier size is 120 nm with a negative surface charge, was effectively loaded with the drug combination, and maintained colloid stability after administration. Mice treated with the nanoparticle showed higher drug accumulation and slower tumor growth than free drug administration [56].

Immunotherapy is another strategy for cancer treatment, in which immune cells are activated or enhanced in their ability to detect and kill cancer cells. One group explored the use of ionizable lipid nanoparticles to deliver mRNA for chimeric antigen receptor (CAR) T cell therapy [57]. CAR is an FDA-approved treatment for acute lymphoblastic leukemia (ALL), in which T cells are collected from the patient and are engineered by introducing DNA or RNA to produce CARs on their surfaces. CAR T cells can recognize and attack cells with the targeted antigen on their surfaces. When lipid nanoparticles comprised of several ionizable lipids were compared to the traditional RNA delivery method of electroporation in delivering mRNA to Jurkat cells, they exhibited lower cytotoxicity while achieving a high transduction ratio, and both CAR T cell engineering methods elicited potent cancer-killing activity [57].

Magnetic hyperthermia is also applied in anti-tumor therapy by targeting magnetic nanoparticles (MNPs) to the tumor site. In this paper, a kind of leukemia targeting MNPs was developed by immobilizing the epithelial cellular adhesion molecule (EpCAM) antibody on the surface of MNPs (EpCAM-MNPs). EpCAM-MNPs can target and remove leukemia cells circulating in the bloodstream and were reported to decrease viability of human monocytic leukemia (THP1) cells by over 40% [58].

### 3.2. Lymphoma 

Lymphoma is a type of cancer that originates from the lymphatic system, enlarging the lymph nodes and metastasizing to other tissues via the lymphatic fluid. After leukemias and brain tumors, lymphoma is the third most common form of cancer among children. According to the National Cancer Institute, around 2200 people under the age of 20 are diagnosed with lymphoma in the United States every year. Hodgkin lymphoma and non-Hodgkin lymphoma comprise approximately 15% of all childhood malignancies [59]. The anti-CD30 antibody-drug conjugate brentuximab and anti-CD20 antibody rituximab are used regularly in adults with lymphoma. However, no targeted agents have been approved for use in pediatric patients with lymphoma [59]. 

Anaplastic large cell lymphoma (ALCL), the most common T-cell pediatric lymphoma, has an active pathogenic ALK oncogene and shows a high level of cell surface expression of CD30. Zeng et al. reported a precision therapy for ALCL using nanoparticles made with RNA-based CD30-specific aptamers (Aptamer—synthetic oligonucleotide or a peptide chain which attaches to a specific target) and loaded with ALK oncogene-specific siRNA and doxorubicin. The conjugated aptamers allowed the nanoparticles to specifically target ALCL cells, while the loaded gene therapy agent siRNA and the chemotherapy agent doxorubicin enhanced their cancer-killing ability [60].

The overactivation of the PI3K/mTOR signaling pathway in non-Hodgkin lymphoma made the corresponding inhibitor BEZ235 a very promising treatment. However, it was withdrawn from early phase clinical trials due to its off-target toxicity and poor solubility. To solve these problems, adding specific antibodies on nanoparticles as targeted drug delivery was a successful strategy. Kin Man Au et al. developed a nanoparticle conjugated with two antibodies, anti-CD20 and anti-Lym1, as tumor-targeting components and loaded them with the PI3K/mTOR inhibitor BEZ235 to treat non-Hodgkin lymphoma [61]. Dual antibody conjugation effectively raised the number of nanoparticles retained on target tumor cells and strengthened the anti-tumor activity of BEZ235 in vitro as well as in vivo models. This is one example of how a nanoparticle-based drug delivery system improves the therapeutic window of small-molecule drugs with substantial on- or off-target toxicity.

In addition to the delivery of small-molecule chemotherapy agents, using nanoparticles to deliver nucleic acid has been explored as an immediate treatment for cancer cells. A recent study shows the lipid nanoparticle-based delivery of siRNA as interference therapy to attack mantle cell lymphoma by silencing their mRNA associated with cancer cell proliferation [62]. To overcome the compensatory upregulation of the cell cycle regulator cyclin D2 that results from the silencing of cyclin D1 by delivering siRNA, two more target molecules, Bcl-2 and Mcl-1, which prevent apoptosis, were treated with these nanoparticles to encapsulate the corresponding siRNA. JeKo-1 cells showed a 75% apoptosis rate and slower dividing time after the nano-cocktail treatment, demonstrating effective siRNA delivery by the nanoparticle [62].

## 4. Bone Cancers

### 4.1. Osteosarcoma

Bone cancers occur mostly in older children and teens and account for about 3% of all pediatric cancers [10]. Osteosarcoma is the most common malignancy of the bone tissue and disproportionately affects pediatric patients. It represents 2% of all pediatric cancers and most commonly affects young adults between the ages of 10 and 30 [63]. Most osteosarcomas in this population are high-grade malignant tumors associated with a poor prognosis [63]. For example, according to data from the American Cancer Society, a distant tumor that has spread beyond nearby tissue is associated with a 27% 5-year survival rate across all ages [64]. As osteosarcoma is the most aggressive bone cancer, it accounts for 9% of pediatric cancer deaths. 

The current standard treatment incorporates presurgical chemotherapy to shrink tumors, surgical resection, radiation therapy to remove what cannot be surgically resected, and post-surgery chemotherapy to lower the chance of relapse [65]. However, because surgery is less effective against advanced osteosarcoma, and multidrug resistance makes treatment challenging, new multimodal therapies are being explored. 

As with other cancer treatments, nanocarrier delivery to osteosarcoma tumors is an emerging research field aimed at increasing targeted drug delivery and decreasing the necessary dosage and cellular toxicity. This is achieved either through passive delivery, relying on the EPR effect, or active delivery, taking advantage of the osteosarcoma cancer environment’s acidic pH and nanoparticle surface modification.

Liposomes have been the most broadly studied vehicle to target osteosarcoma due to their biocompatibility and surface modification ability [66]. Specifically, liposomes have been loaded with doxorubicin and have shown increased cell permeability and tumor cell death compared to free doxorubicin [67]. Some studies have shown success in optimizing liposome nanocarriers to release the drug in the specific temperature and pH of the osteosarcoma tumor [68]. Others have explored the PEGylation of liposomes, which diminishes nanoparticle re-uptake by the reticuloendothelial system, leading to a longer half-life and lower optimal dosage [69]. Recently, an in vitro study demonstrated synergistic benefits of gemcitabine and clofazimine when dually loaded into nanoparticles. This dual loading was achieved by loading hydrophobic gemcitabine into the liposome core and hydrophilic clofazimine between the lipid bilayers [70].

RNAi therapies such as microRNAs and siRNAs have shown promise in downregulating proteins produced by osteosarcoma cells and could be effective in conjunction with chemotherapies [71,72]. However, potential carriers of nucleic acids are still a barrier to progress, as their poor physicochemical characteristics limit bioavailability and cell uptake. Some research has been published on possible biocompatible carriers, such as Amy-g-PLLD [73]. PEGylated liposomes have been studied for the delivery of siRNA, both alone and with doxorubicin [74,75]. In 2017, one study reported enhanced tumor cell uptake, anti-tumor effects, and improved survival rate in murine models using chitooligosaccharides to enhance drug delivery [76].

### 4.2. Ewing Sarcoma 

Ewing sarcoma is another common malignant bone tumor that primarily affects adolescents and young adults. While it most frequently presents as a bone tumor, it can also develop in connective tissue and soft tissue surrounding bone. Surgery is the most common method for removing Ewing sarcoma, while chemo- and radiation therapy are usually performed to shrink the tumor before surgery or prevent metastasis and recurrence after surgery. Current protocols include five chemotherapeutic agents (cyclophosphamide, topotecan, etoposide, doxorubicin, and ifosfamide), four of which induce DNA damage, as does radiation therapy [77,78,79,80,81]. At relapse, two additional DNA-damaging agents (irinotecan and temozolomide) are routinely used to re-induce remission. However, the incidence of Ewing sarcoma has remained unchanged for 30 years [82,83], and there is no treatment available for patients who relapse. The relapse rate in Ewing sarcoma patients is also higher than for any other pediatric cancer, with the 5-year event-free survival rate for such patients at only about 20%. 

Some research has suggested that using liposomes to deliver chemotherapy for Ewing sarcoma can decrease toxicity and increase drug circulation. In one report, nanoliposome formulas encapsulating the PARP1 inhibitor talazoparib increased the tolerated dose compared to oral administration [84,85]. Another study found that liposomes formulated from PLGA increased the half-life of docetaxel [86]. Bisphosphonates such as zoledronic acid can inhibit cancer angiogenesis, and because of their affinity to bone, nanoparticles conjugated with bisphosphonates showed an increased uptake and cell toxicity compared to pegylated PGLA nanoparticles [87,88]. Fontaine et al. showed that long-acting PEGylated talazoparib has promising anti-tumor activity in Ewing sarcoma [89]. One approach to Ewing sarcoma involves silencing the miRNA that drives CD99, a hallmark surface antibody in the disease. Exosomes from CD-99-deprived Ewing sarcoma cells can act as “natural” targeted nanocarriers of chemotherapy [90].

The simultaneous delivery of two or more drugs that may further sensitize cancer cells (an approach called synthetic lethality) is also a strategy for cancer treatment and recurrence prevention. The Pediatric Preclinical Testing Program (PPTP) identified this synergistic activity by using talazoparib plus temozolomide to treat Ewing sarcoma [85]. However, the severe toxicity of this combination limits its clinical application. Baldwin et al. reported that a nanoformulation of talazoparib plus temozolomide reduced gross toxicity and resulted in a higher tolerated dose than oral talazoparib combined with temozolomide [84]. 

Another study tested the use of a hydrolyzed galactomannan (hGM)-based amphiphilic nanoparticle for selective intratumoral accumulation in pediatric sarcoma [91]. This self-assembled nanoparticle was created by linking the side chain of hGM with poly (methyl methacrylate) through a graft free radical polymerization reaction, encapsulated with tyrosine kinase inhibitor imatinib with a 7.5% of efficiency. The findings suggest that these nanoparticles can target GLUT-1, as the internalization ratio was 100% in rhabdomyosarcoma and Ewing sarcoma.

Alhaddad et al. reported the use of a novel diamond nanoparticle coated with cationic polymers to deliver interfering RNA to Ewing sarcoma cells [92]. In this strategy, siRNA is absorbed into diamond nanocrystals, and cell uptake is imaged using the nanocrystals’ intrinsic fluorescence caused by embedded color-center defects. The cell toxicity of these coated NDs is shown below. The diamond nanocrystal-vectorized siRNA specifically inhibited expression of *EWSR1-FLI1* at the mRNA and protein levels in a serum-containing medium. Diamond nanocrystals also display fluorescence properties that result from the creation of a nitrogen-vacancy color center inside the nanodiamond matrix. This property means they could be used for tracking throughout the lifespans of cells and organisms.

Lipoproteins are also appropriate materials for nanoparticle synthesis. Bell et al. employed biomimetic high-density lipoprotein (HDL) nanoparticles. These bind to HDL receptors and scavenger receptor type B-1 (SCARB1), which in turn deprives cells of natural HDL and their cholesterol stores, and blocks cell proliferation. In medulloblastoma and hedgehog-driven Ewing sarcoma tissues, this strategy depleted the populations of cancer stem cells. Furthermore, HDL nanoparticles disrupted cell colony formation in medulloblastomas [93]. This study suggests that HDL-mimetic nanoparticles are a promising therapy for the sonic hedgehog subtype of medulloblastoma. 

## 5. Cancers of the Central Nervous System

### 5.1. Brain Cancer

Brain cancers comprise the second most common cancer in children, making up about 26% of all pediatric cancers [10]. Brain cancers are named based on the cell type from which cancer originated and the tumor location in the brain. They are treated with surgery, radiation, and chemotherapy. However, brain tumors can present a challenge for surgery and the delivery of therapeutic agents depending on their location. 

### 5.2. Blood–Brain Barrier 

The blood–brain barrier (BBB) is a dynamic interface that separates the brain from the circulatory system to protect the brain from potentially harmful chemicals and pathogens and regulate the transport of essential nutrition to maintain a stable microenvironment [94]. The BBB is made up of continuous endothelial cells closely sealed by tight junctions and surrounded by astrocytes, pericytes, and the continuous basement membrane. It is a highly selective semipermeable border with a high expression of distinct sets of transporter proteins that only permit the free diffusion of essential small molecules like oxygen. The effectiveness of the BBB means that fewer drugs can be efficacious and the prognosis of pediatric patients with brain cancer is worse [95].

Overwhelmingly, the literature suggests that molecular size plays a vital role in BBB penetration. However, molecular size does not necessarily interfere with BBB permeability [94]. Some small molecules with a molecular weight of around 100 Da, like histamine, do not enter the brain due to the BBB [96]. However, if candidate agents interact with the major transporters on the BBB, it can help them pass through it. The inability of many drugs to reach the brain is attributed to the activity of the ATP-binding cassette (ABC) efflux transporters, such as the breast cancer resistance protein (BCRP) [94].

Nanotechnology has enabled significant progress in delivering therapeutic agents across the BBB. Materials such as gold, lipids, and proteins are being investigated as cytostatic agents or drug carriers to treat or diagnose brain cancer. Nanoparticle size and surface are two main factors that affect the ability to cross the BBB. Large particles (over 150 nm in diameter) tend to be blocked by it, whereas a slightly positive surface charge can be favorable for particle–endothelial cell binding [95]. Antibodies with a molecular weight larger than 500 kDa, such as intact IgG typically used to treat various types of cancers, show a low penetration of the BBB [97]. Smaller antibodies such as single-chain variable fragments or fragment antigen-binding (Fab) may improve penetration into the central nervous system.

Apart from diffusion, ligand-induced transcytosis is also being tested for delivering drugs across the BBB. While BBB is a highly selective semipermeable endothelial cell border, the unique protein transporters expressed on its membrane that allow necessary nutrients to maintain brain homeostasis provide ideal targets. 

The transferrin receptor (TfR) is responsible for transporting iron into the brain parenchyma to maintain appropriate iron levels needed for brain metabolism, neural conductivity, and overall brain function [98]. The TfR is an intriguing and unique target since it is exclusively expressed on the endothelial cells of the brain capillaries and not on endothelial cells lining the vessels in other tissues [99,100]. This specific property makes transferrin receptor antibody an intriguing concept for delivering drugs through the BBB. When conjugated to nanoparticles, such as liposomes, BBB targeting and penetration will be significantly improved [101,102,103,104]. 

Similarly, cell-penetrating peptides (CPPs), which facilitate the cellular uptake of molecules ranging from nano-size particles to small chemical compounds to large fragments of DNA, have been used to penetrate the BBB. Wang et al. tested the efficacy of CPPs and transferrin modified liposomes (Tf-LPs) loaded with doxorubicin to treat glioma. This nanoparticle, which was 120 nm in size and had a zeta potential of 6.81 mV, showed enhanced cellular uptake and reduced toxicity in two types of glioma cells compared to free doxorubicin [105].

Another promising marker for malignant glioma is the amplification of the epidermal growth factor receptor (EGFR) expression with a frequency of about 50% [106,107]. ErbB1 belongs to the ErbB family of receptor tyrosine kinases, including human epidermal growth factor receptors (HER)-2/ErbB2, HER-3/ErbB3, and HER-4/ErbB4 [108]. The application of EGF, the natural ligand of EGFR, is a potential strategy to target every subset of tumor cell expressing wild-type EGFR as well as its mutant forms. The conjugation of EGF to the nanoparticle could enable targeted treatment for glioma [109,110].

Immunotherapy is more commonly used for brain tumors than for other tumor types because of the brain’s unique environment, which can be regarded as an immune-privileged site separate from the rest of the body that prohibits immune cells from entering [97,111,112,113]. Therefore, the microglia take a predominant position inside the brain and tend to be pro-tumorigenic under certain conditions, such as the intensive secretion of growth factors and lack of appropriate T-cell regulation. Moreover, most brain tumors have a unique extracellular structure that provides the inhibitory regulation of T cells to prevent their migration and activation [114]. This suggests that immunotherapy should be adjusted based on the specific cancer type or even certain gene mutations and their surrounding microenvironments. Specifically, the selected tumor antigens should have a tumor-unique expression pattern, the ability to activate T cells and elicit the ensuing immune response, and ideally should downregulate tumor functions to minimize tumors’ ability to bypass the immune system. Short peptides or antibodies are the most frequently used immuno-stimulating agents for this purpose due to their specific binding capacity [115]. They are commonly used together with a drug delivery platform (nanoparticles loaded with a combination of drugs) as a dual treatment method [116,117,118]. 

### 5.3. Glioma

Glioma is one of the most common brain cancers in children and adolescents, and originates from glial cells, which support and nourish nerves in the brain [119]. A folacin-modified poly(e-caprolactone) micelle was designed to deliver luteolin—a xanthone extracted from vegetables with broad spectra anti-cancer effects—to treat glioblastoma [120]. Folate acids were conjugated onto the surface of this nanoparticle to allow the binding to the folate receptor, a type of glycoprotein with increased expression in many tumor tissues. Compared to free luteolin and micelles without folacin modification, luteolin-loaded folate acid-modified micelles to glioma tissues induces a significantly higher cell inhibition and increased apoptosis in glioma [120]. 

**Figure 2:**
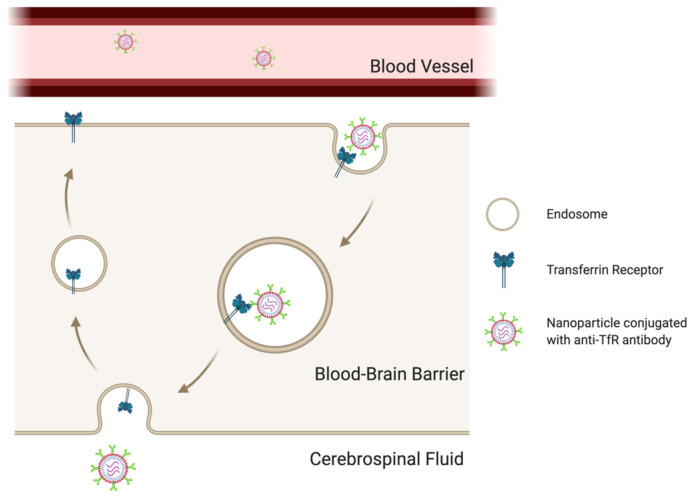
Transferrin receptor (TfR)-mediated transcytosis across the blood–brain barrier (BBB). The transcytosis is initiated by the binding of anti-TfR antibody conjugated on the surface of nanoparticles with the formation of endosome. Nanoparticles are transported to the brain side by membrane fusion. TfRs are then recycled to the original side also by the transport of endosome. (Created with BioRender.com at 6 March 2021).

TfRs are overexpressed in both BBB endothelial cells and gliomas. Fan et al. reported a trans-BBB delivery strategy that used Human H-Ferritin and L-Ferritin protein-covered iron oxide nanoparticles (HFns) to target the TfRs of the BBB endothelial cells and induce transcytosis [121] (Figure 2). The nanoparticles demonstrated adequate loading capacity for various drugs and excellent dual tumor-targeting prospects. They were carried through the BBB in the endosome by TfR-mediated transcytosis, recognizing and entering the glia cells by human H-ferritin receptor-mediated tumor targeting. 

### 5.4. Medulloblastoma

Medulloblastoma is a common pediatric brain tumor located in the cerebellum, the lower back part of the brain that controls coordination, movement, and balance. Once established, medulloblastoma tends to spread to other parts of the brain through the cerebrospinal fluid, but rarely spreads to other body tissues. It occurs at any age, but most likely to happen in childhood, and is rare in adults. 

Choi et al. explored the strategy of suicide gene therapy for pediatric brain cancer medulloblastoma. Poly(beta-amino ester) nanoparticles were developed to deliver plasmid DNA encoding the suicide gene of herpes simplex virus I thymidine kinase [122]. The delivery of the virus suicide gene induced the controlled apoptosis of transfected cancer cells and prolonged overall survival in mice. These results suggest that these biodegradable nanoparticles could be a safe and effective method for treating pediatric CNS malignancies.

Kim et al. engineered high-density lipoprotein-mimetic nanoparticles with an enhanced stability and targeting ability for treating the sonic hedgehog (SHH) subtype of medulloblastoma [123]. Apolipoprotein A1 was incorporated into the shell of the nanoparticle and provided better structural stability while keeping LDE225, an SHH inhibitor, encapsulated in the hydrophobic core of the nanocarrier. Anti-CD15 was conjugated on the surface of engineered high-density lipoprotein-mimetic nanoparticles (eHNPs) for receptor-facilitated delivery. eHNPs can serve as stable drug carriers while also providing a therapeutic effect through SR-B1-mediated intracellular cholesterol deletion in SHH medulloblastoma.

Radiotherapy is an integral component of cancer treatment. However, the radiation-induced adverse sequelae and resistance create clinical limitations. Thus, combining radiotherapy with different therapeutic agents that block specific DNA repair pathways could achieve a better therapeutic efficacy than monotherapy with a lower radiation dosage that minimizes potential adverse effects. Kievit et al. reported a strategy to sensitize pediatric tumor cells, including medulloblastoma and ependymoma cells, to radiotherapy by the nanoparticle-based delivery of siRNA; this knocks down the expression of Ape1, an enzyme involved in the base excision repair pathway [124]. This superparamagnetic iron oxide nanoparticle was coated with chitosan, PEG, and polyethyleneimine and can bind to siRNA and protect it from degradation. The treated medulloblastoma and ependymoma cells exhibited over 75% reduction in Ape1 expression and 80% inhibition of Ape1 activity, which indicates the potential for siApe1 as an efficacious delivery strategy.

## 6. Less Common Cancers 

### 6.1. Retinoblastoma 

Retinoblastoma makes up 3% of all childhood cancers, and in severe cases, it can cause blindness and metastasis beyond the eye if not treated promptly and effectively [125,126]. Ocular malignancies pose unique challenges, and monotherapies must provide enhanced permeation through the retinal pigment endothelial layer [127]. A variety of nano-applications have been explored in recent years to surpass these challenges. One study found that introducing biodegradable nanoparticles into tumor tissues during laser irradiation treatment allowed for selective damage to retinal cancer cells by decreasing the heat capacity and increasing the thermal conductivity of cancerous tissue. Sensitizing the cancerous tissue refined the treatment and increased the lethal zone area by 51% [128]. A similar study found that the cytotoxicity of retinoblastoma cells treated with ultrasonic hyperthermia increased with the concentration of gold nanoparticles present. The same cell viability (50%) was achieved in half the time when gold nanoparticles were present. [129] Another study evaluated the efficacy of injecting conjugated gold nanorods into the eye (using femtosecond pulse lasers) to selectively accumulate in retinoblastoma cells and induce the ablation of only those cells containing the nanorods. In combination, gold nanorods and femtosecond pulse lasers decreased the viability of retinoblastoma cells to about 10%, compared to 100% viability in untreated cells [130]. 

Researchers explored the delivery of natural therapeutics through nanoparticles to treat retinoblastoma. One study utilized polymeric nano-micelles as a delivery method to improve the water solubility of celestrol, a Chinese herb that displays the inhibition of angiogenesis-mediated retinoblastoma growth in murine models [131]. Similar to many other pediatric cancers, nanoparticles have been used for drug delivery to reduce the cytotoxicity of chemotherapies. A study explored the targeted co-delivery in lipid nanoparticles of miR-181a, a microRNA, and melphalan, a currently approved chemotherapy for retinoblastoma. The nanoparticles were outfitted with a cationic lipid that changed the conformation at the acidic pH of retinoblastoma tissue to trigger endosomal release, as well as several structural lipids to improve the structure, fluidity, and colloidal stability. In vivo, the combination therapy reduced retinoblastoma cells by 72% compared to treatment with free melphalan alone [132].

### 6.2. Wilms Tumor

Wilms tumor is the most common form of pediatric kidney cancer and accounts for 5% of all pediatric cancers [133]. Nanomedicine applications are sparse in Wilms tumor. One in vitro study targeted neural cell adhesion molecule (NCAM) expressed by Wilms tumor stem cells with a nanosized conjugate of paclitaxel bound to a biodegradable polyglutamic acid polymer. The conjugate reduced tumor size by roughly five times compared to untreated cells [134].

### 6.3. Other Pediatric Cancers

Nanomedicine is still a relatively new field, and new oncology applications are being developed at exponential rates. The applications discussed above could be modified to treat several rare yet consequential pediatric cancers. Hepatoblastoma, hepatocellular carcinoma, pleuropulmonary blastoma, and tracheobronchial tumor together account for less than 2% of all pediatric cancers and require further exploration into nano-applications and diagnostics and treatment.

## 7. Summary and Future Perspective

With the maturity of novel anti-cancer therapy, more and more nanoparticle-based drugs are also being approved by the FDA, improving outcomes for adult cancer patients. Nonetheless, effective strategies for treatment-resistant pediatric cancers remain elusive and only a few drugs are now approved for pediatric patients. Currently, pediatric cancer is commonly treated with chemotherapy, which triggers potential severe side-effects and causes toxicity to normal tissues. With different metabolic rates and immature organs, the tolerated dose is also different in children and adults, making it more challenging to identify the optimal dosage. To prevent the drug resistance often brought about by monotherapy, the combination of two or more chemotherapy agents changed little over the past two decades. However, organs and tissues grow rapidly in children, which means that they can respond differently to the medication at different developmental stages. As a result, pediatric cancer survival rates remain low. What is worse, a large portion of survivors suffer from short- or long-term adverse effects, such as nephrotoxicity, cardiotoxicity, infertility, and deafness. Targeted therapy is studied extensively and believed to be a promising strategy to overcome off-target toxicity associated with such diseases. Nanoparticles can easily achieve this goal by conjugating the antibodies or specific peptides against the target proteins. This emerging field is of high interest to researchers in academia and pharmaceutical companies, leading to tremendous development progress. All of the nanoformulations discussed in this review are shown in Table 2. However, most novel nanomedicine milestones have been achieved in the adult cancer field, while pediatric cancer nanotherapy is still in its early stage. For example, the biocompatibility of several liposome formulations is well known, and many nanodrug delivery systems have reached the clinical phase in adults, however, the information regarding their safety in children is very limited [135]. The main obstacles for pediatric nanomedicine development are the current dearth of clinical trial protocols that we discussed above. Nevertheless, as nanotechnology becomes more broadly accepted and more nanotechnology-related products are developed, the greater use of nanomedicine applications (also as part of the RACE for Children Act) for childhood cancer therapy is expected in the near future.

## Figures and Tables

**Table 1:** Liposome formulations under clinical trial for pediatric cancer *.

Phase	Drug Loaded	Study Start Date	Study Completion Date	Recruitment Status	Last Update Posted	Ages Eligible for Study	ClinicalTrials.gov Identifier	Type of Cancer
Phase 1	Irinotecan	December 2013	December 2020	Recruiting	18 September 2019	1 to 20	NCT02013336	ST, ES, RhS, NB, OS
Phase 1	Doxorubicin	October 2016	October 2021	Recruiting	25 September 2020	Up to 30 years	NCT02536183	ST, RhS, ES, STS, OS, NB, WT, HT, GCT
Phase 1	doxorubicin	July 1999	-	Completed	28 April 2015	Up to 21 years	NCT00019630	STS, LC, BC, BT, KT
Phase 1	Doxorubicin	December 2016	16 March 2019	Withdrawn	19 March 2019	1 year to 40 years	NCT02557854	RhS, NB, ES, OS
Phase 1	cytarabine	February 1997	-	Unknown	23 March 2010	1 year to 21 years	NCT00003073	CNST, Leukemia, Lymphoma
Phase 2	Daunorubicin	13 March 2019	12 May 2027	Suspended	13 February 2020	3 months to 17 years	NCT03591510	FLT3-mutated AML
Phase 2	Daunorubicin	6 August 2019	June 2022	Recruiting	24 November 2020	Up to 17 years	NCT03860844	ALL, AML
Phase 2	Cytarabine	January 2013	December 2019	Recruiting	13 March 2019	3 years to 31 years	NCT01859819	DLCL, BL, HGBL
Phase 2	Cytarabine	January 1996	June 2004	Completed	1 February 2013	Up to 20 years	NCT00002704	Leukemia
Phase 2	Vincristine	June 2000	September 2005	Completed	31 October 2018	Child, adult, older adult	NCT00038207	STS, WT, OS, Lymphoma, Leukemia
Phase 2	Vincristine	23 November 2016	11 March 2018	Terminated	3 April 2019	Up to 21 years	NCT02518750	ALL, NHL, Leukemia

* Data collected on 5 December 2020.

**Table 2:** Nanoformulations discussed in this review.

Formulation	Drug Loaded	Targeting Anent	Size (nm)	ZP (mV)	Diseases	Cancer/Animal Model	Route	Reference
Solid lipid nanoparticle	Mitoxantrone β-element	-	124.6	0.162	Leukemia	K562/DOX xenografts tumor model mice	IV ^1^	[56]
Ionizable lipid nanoparticle	mRNA	-	70	-	Leukemia	Human Jurkat cell line	-	[57]
Magnetic nanoparticles	Hyperthermia effect	Epithelial cellular adhesion molecule	5	-	Leukemia	AKR mice	IP ^2^	[58]
Aptamer-equipped protamine nanoparticle	dsDNA/doxorubicin complex, siRNAs	Oligonucleotide aptamers	103	-	Lymphoma	Human ALCL cell lines	-	[60]
PEG-PLGA nanoparticle	BEZ235	Anti-CD20, anti-Lym1	70	-	Lymphoma	CD-1 mice	IV	[61]
Lipid nanoparticle	siRNA	-	100	-	Lymphoma	JeKo-1/MAVER-1 human mantle cell lymphoma cell lines	-	[62]
Liposome	Doxorubicin	-	93.61	−23	Osteosarcoma	Human MG-63 cell line	-	[68]
PEGylated-liposome	Doxorubicin	-	-	-	Osteosarcoma	Phase II trial	IV	[69]
Liposome	Gemcitabine, clofazimine	-	135	−9.3	Osteosarcoma	Human Saos-2 cell line	-	[70]
Polysaccharide nanoparticle	siRNA	Folic acid	270	10	Osteosarcoma	Osteosarcoma 143B cells. Tumor-bearing mice models	IV	[73]
PEGylated liposome	siRNA	-	100	19.24	Osteosarcoma	Human MG-63 cell line	-	[74]
PEGylated liposome	Doxorubicin, JIP1 siRNA	YSA peptide	108.9	18.47	Osteosarcoma	Human SaOs-2/MG-63 cell lines	-	[75]
Chitooligosaccharides modified liposome	Doxorubicin	Chitooligosaccharides	100	33.9	Osteosarcoma	MG63 cell-bearing nude mice	IV	[76]
Liposome	Talazoparib	-	74.5	15.3	Ewing sarcoma	NCr-nu/nu and scid CB17 mice	IV	[84]
Galactomannan-based nanoparticle	Imatinib	Monosaccharide and disaccharide residues	84	−0.5	Ewing sarcoma, rhabdomyosarcoma	Mice bearing PDX models	IV	[91]
Diamond nanoparticle	siRNA	-	50	27	Ewing sarcoma	Ewing sarcoma mouse model	IV	[92]
High-density lipoprotein nanoparticle	High-density lipoprotein	High-density lipoprotein	10	-	Medulloblastoma, Ewing sarcoma	Human DAOY/D283 cell line	-	[93]
Liposome	Doxorubicin	Cell-penetrating peptide, transferrin	128.64	6.81	Glioma	Intracranial U87 glioma-bearing mice	IV	[105]
PEGlyated micelle	Luteolin	Folic acid	34.7	−9.2	Glioma	C57 mice	IV	[120]
H-ferritin nanoparticle	Doxorubicin	H-ferritin	12	-	Glioma	U87MG orthotopic tumor-bearing mice	IV	[121]
PBAE nanoparticle	Plasmid DNA	-	100–200	12	Medulloblastoma	Athymic nude mice with 5e5 BT-12 cells	IP	[122]
High-density lipoprotein nanoparticle	LDE225	Apolipoprotein A1, anti-CD15	15	-	Medulloblastoma	SmoA1+/+: Math1-GFP+/+ SmoA1 MB tumor-bearing mice	IV	[123]
PEGylated iron oxide nanoparticle	siRNA	-	40	15	Medulloblastoma, ependymoma	Human UW228-1/Res196 cell line	-	[124]
Magnesium oxide nanoparticle	Hyperthermia effect	-	-	-	Retinoblastoma	Predictive finite element cancerous human eye model	-	[128]
Gold nanoparticle	Ultrasound hyperthermia	-	89	38.6	Retinoblastoma	Human Y79 cell line	-	[129]
PEGylated gold nanoparticle	-	Anti-EpCAM	11	-	Retinoblastoma	Squamous cell carcinoma xenografts in nu/nu mice	IV	[130]
Celastrol nanomicelle	Celastrol	-	48	12	Retinoblastoma	Female NOD-SCID mice	IP	[131]
Lipid nanoparticles	miR-181a, melphalan	-	171	24.5	Retinoblastoma	Human Y-79 cell line	-	[132]
PGA nanoparticle	Paclitaxel	NCAM targeting peptide	10	-	Wilms Tumor	NOD/SCID mice	IV	[134]

^1^ IV: intravenous administration; ^2^ IP: intraperitoneal administration.

## Abbreviations

ST	Solid tumor
ES	Ewing sarcoma
RhS	Rhabdomyosarcoma
NB	Neuroblastoma
OS	Osteosarcoma
STS	Soft tissue sarcomas
WT	Wilms tumor
HT	Hepatic tumor
GCT	Germ cell tumors
LC	Liver cancer
BC	Bone cancer
BT	Brain tumor
KT	Kidney tumor
CNST	Central nervous system tumor
ALL	Acute lymphoblastic leukemia
NHL	Non-Hodgkin’s lymphoma
DLCL	Diffuse large cell lymphoma
BL	Burkitt’s lymphoma
AML	Acute myeloid leukemia
HGBL	High grade B-cell lymphoma

## References

[1] American Cancer Society (2021). Key Statistics for Childhood Cancers. https://www.cancer.org/cancer/cancer-in-children/key-statistics.html.

[2] Lam C.G., Howard S.C., Bouffet E., Pritchard-Jones K. (2019). Science and health for all children with cancer. Science.

[3] Smith M.A., Altekruse S.F., Adamson P.C., Reaman G.H., Seibel N.L. (2014). Declining childhood and adolescent cancer mortality. Cancer.

[4] Colletti M., Paolo V.D., Galardi A., Milano G.M., Mastronuzzi A., Locatelli F., Di Giannatale A. (2017). Nano-Delivery in Pediatric Tumors: Looking Back, Moving Forward. Anti Cancer Agents Med. Chem..

[5] Pritchard-Jones K., Pieters R., Reaman G.H., Hjorth L., Downie P., Calaminus G., Naafs-Wilstra M.C., Steliarova-Foucher E. (2013). Sustaining innovation and improvement in the treatment of childhood cancer: Lessons from high-income countries. Lancet Oncol..

[6] Kuerbanjiang A., Maimaituerxun M., Zhang Y., Li Y., Cui G., Abuduhabaier A., Aierken A., Miranbieke B., Anzaer M., Maimaiti Y. (2021). V-Raf murine sarcoma viral oncogene homolog B1 (BRAF) as a prognostic biomarker of poor outcomes in esophageal cancer patients. BMC Gastroenterol..

[7] Gerber D.E. (2008). Targeted therapies: A new generation of cancer treatments. Am. Fam. Physician.

[8] Stone W.L., Klopfenstein K.J., Hajianpour M.J., Popescu M.I., Cook C.M., Krishnan K. (2017). Childhood cancers and systems medicine. Front. Biosci. Landmark Ed..

[9] Udaka Y.T., Packer R.J. (2018). Pediatric Brain Tumors. Neurol. Clin..

[10] American Cancer Society Types of Cancer that Develop in Children. https://www.cancer.org/cancer/cancer-in-children/types-of-childhood-cancers.html.

[11] Siegel R.L., Miller K.D., Jemal A. (2020). Cancer statistics, 2020. CA Cancer J. Clin..

[12] Rahal Z., Abdulhai F., Kadara H., Saab R. (2018). Genomics of adult and pediatric solid tumors. Am. J. Cancer Res..

[13] Gröbner S.N., Worst B.C., Weischenfeldt J., Buchhalter I., Kleinheinz K., Rudneva V.A., Johann P.D., Balasubramanian G.P., Segura-Wang M., Brabetz S. (2018). The landscape of genomic alterations across childhood cancers. Nature.

[14] Ma X., Liu Y., Liu Y., Alexandrov L.B., Edmonson M.N., Gawad C., Zhou X., Li Y., Rusch M.C., Easton J. (2018). Pan-cancer genome and transcriptome analyses of 1699 paediatric leukaemias and solid tumours. Nature.

[15] Parham D.M., Barr F.G. (2013). Classification of rhabdomyosarcoma and its molecular basis. Adv. Anat. Pathol..

[16] Sankar S., Lessnick S.L. (2011). Promiscuous partnerships in Ewing’s sarcoma. Cancer Genet..

[17] Ladanyi M. (2001). Fusions of the SYT and SSX genes in synovial sarcoma. Oncogene.

[18] Wang Y., Wu N., Liu D., Jin Y. (2017). Recurrent Fusion Genes in Leukemia: An Attractive Target for Diagnosis and Treatment. Curr. Genom..

[19] Rodríguez-Nogales C., González-Fernández Y., Aldaz A., Couvreur P., Blanco-Prieto M.J. (2018). Nanomedicines for Pediatric Cancers. ACS Nano.

[20] Pearson A.D., Stegmaier K., Bourdeaut F., Reaman G., Heenen D., Meyers M.L., Armstrong S.A., Brown P., De Carvalho D., Jabado N. (2020). Paediatric Strategy Forum for medicinal product development of epigenetic modifiers for children: ACCELERATE in collaboration with the European Medicines Agency with participation of the Food and Drug Administration. Eur. J. Cancer.

[21] American Association for Cancer Research (2020). RACE Act Poised to Advance Pediatric Cancer Research. Cancer Discov..

[22] Hwang T.J., Orenstein L., DuBois S.G., Janeway K.A., Bourgeois F.T. (2020). Pediatric Trials for Cancer Therapies with Targets Potentially Relevant to Pediatric Cancers. J. Natl. Cancer Inst..

[23] Howard S.C., Zaidi A., Cao X., Weil O., Bey P., Patte C., Samudio A., Haddad L., Lam C.G., Moreira C. (2018). The My Child Matters programme: Effect of public-private partnerships on paediatric cancer care in low-income and middle-income countries. Lancet Oncol..

[24] Palacios-Macedo A., Mery C.M., Cabrera A.G., Bastero P., Tamariz-Cruz O., Díliz-Nava H., García-Benítez L., Pérez-Juárez F., Araujo-Martínez A., Mier-Martínez M. (2019). A Novel Private-Public Hybrid Model for Treatment of Congenital Heart Disease in Mexico. World J. Pediatr. Congenit. Heart Surg..

[25] Perry C.L., Hoelscher D.M., Kohl H.W. (2015). Research contributions on childhood obesity from a public-private partnership. Int. J. Behav. Nutr. Phys. Act..

[26] Irvine D.J., Hanson M.C., Rakhra K., Tokatlian T. (2015). Synthetic Nanoparticles for Vaccines and Immunotherapy. Chem. Rev..

[27] Irvine D.J., Dane E.L. (2020). Enhancing cancer immunotherapy with nanomedicine. Nat. Rev. Immunol..

[28] Bonam S.R., Kotla N.G., Bohara R.A., Rochev Y., Webster T.J., Bayry J. (2021). Potential immuno-nanomedicine strategies to fight COVID-19 like pulmonary infections. Nano Today.

[29] Polo E., Kruss S. (2016). Nanosensors for neurotransmitters. Anal. Bioanal. Chem..

[30] Han Q., Niu M., Wu Q., Zhong H. (2018). Real-time Monitoring of Nanoparticle-based Therapeutics: A Review. Curr. Drug Metab..

[31] Chaturvedi V.K., Singh A., Singh V.K., Singh M.P. (2019). Cancer Nanotechnology: A New Revolution for Cancer Diagnosis and Therapy. Curr. Drug Metab..

[32] Baetke S.C., Lammers T., Kiessling F. (2015). Applications of nanoparticles for diagnosis and therapy of cancer. Br. J. Radiol..

[33] Blanco E., Shen H., Ferrari M. (2015). Principles of nanoparticle design for overcoming biological barriers to drug delivery. Nat. Biotechnol..

[34] Hoshyar N., Gray S., Han H., Bao G. (2016). The effect of nanoparticle size on in vivo pharmacokinetics and cellular interaction. Nanomedicine.

[35] Pérez-Herrero E., Fernández-Medarde A. (2015). Advanced targeted therapies in cancer: Drug nanocarriers, the future of chemotherapy. Eur. J. Pharm. Biopharm..

[36] Kang H., Rho S., Stiles W.R., Hu S., Baek Y., Hwang D.W., Kashiwagi S., Kim M.S., Choi H.S. (2020). Size-Dependent EPR Effect of Polymeric Nanoparticles on Tumor Targeting. Adv. Healthc. Mater..

[37] Mita M.M., Mita A.C., Moseley J.L., Poon J., Small K.A., Jou Y.M., Kirschmeier P., Zhang D., Zhu Y., Statkevich P. (2017). Phase 1 safety, pharmacokinetic and pharmacodynamic study of the cyclin-dependent kinase inhibitor dinaciclib administered every three weeks in patients with advanced malignancies. Br. J. Cancer.

[38] Maeda H. (2001). The enhanced permeability and retention (EPR) effect in tumor vasculature: The key role of tumor-selective macromolecular drug targeting. Adv. Enzym. Regul..

[39] Baldwin P., Ohman A.W., Medina J.E., McCarthy E.T., Dinulescu D.M., Sridhar S. (2019). Nanoformulation of Talazoparib Delays Tumor Progression and Ascites Formation in a Late Stage Cancer Model. Front. Oncol..

[40] Alavi M., Hamidi M. (2019). Passive and active targeting in cancer therapy by liposomes and lipid nanoparticles. Drug Metab. Pers. Ther..

[41] Swain S., Sahu P.K., Beg S., Babu S.M. (2016). Nanoparticles for Cancer Targeting: Current and Future Directions. Curr. Drug Deliv..

[42] Muhamad N., Plengsuriyakarn T., Na-Bangchang K. (2018). Application of active targeting nanoparticle delivery system for chemotherapeutic drugs and traditional/herbal medicines in cancer therapy: A systematic review. Int. J. Nanomed..

[43] Hernández-Muñoz P., Cerisuelo J.P., Domínguez I., López-Carballo G., Catalá R., Gavara R., López Rubio A., Fabra Rovira M.J., Martínez Sanz M., Gómez-Mascaraque L.G. (2019). Chapter 8—Nanotechnology in Food Packaging. Nanomaterials for Food Applications.

[44] Farooq M.U., Novosad V., Rozhkova E.A., Wali H., Ali A., Fateh A.A., Neogi P.B., Neogi A., Wang Z. (2018). Gold Nanoparticles-enabled Efficient Dual Delivery of Anticancer Therapeutics to HeLa Cells. Sci. Rep..

[45] Feng T., Wei Y., Lee R.J., Zhao L. (2017). Liposomal curcumin and its application in cancer. Int. J. Nanomed..

[46] Zhang Y., Huang Y., Li S. (2014). Polymeric micelles: Nanocarriers for cancer-targeted drug delivery. Aaps Pharmscitech.

[47] Vangijzegem T., Stanicki D., Laurent S. (2019). Magnetic iron oxide nanoparticles for drug delivery: Applications and characteristics. Expert Opin. Drug Deliv..

[48] Ayyanaar S., Kesavan M.P., Balachandran C., Rasala S., Rameshkumar P., Aoki S., Rajesh J., Webster T.J., Rajagopal G. (2020). Iron oxide nanoparticle core-shell magnetic microspheres: Applications toward targeted drug delivery. Nanomed. Nanotechnol. Biol. Med..

[49] Zhu L., Zhou Z., Mao H., Yang L. (2017). Magnetic nanoparticles for precision oncology: Theranostic magnetic iron oxide nanoparticles for image-guided and targeted cancer therapy. Nanomedicine.

[50] Kievit F.M., Zhang M. (2011). Surface engineering of iron oxide nanoparticles for targeted cancer therapy. Acc. Chem. Res..

[51] Chee C.F., Leo B.F., Lai C.W., Inamuddin Asiri A.M., Mohammad A. (2018). 37—Superparamagnetic iron oxide nanoparticles for drug delivery. Applications of Nanocomposite Materials in Drug Delivery.

[52] Arias L.S., Pessan J.P., Vieira A.P.M., Lima T.M.T., Delbem A.C.B., Monteiro D.R. (2018). Iron Oxide Nanoparticles for Biomedical Applications: A Perspective on Synthesis, Drugs, Antimicrobial Activity, and Toxicity. Antibiotics.

[53] Bianco A., Kostarelos K., Prato M. (2005). Applications of carbon nanotubes in drug delivery. Curr. Opin. Chem. Biol..

[54] Elhissi A.M., Ahmed W., Hassan I.U., Dhanak V.R., D’Emanuele A. (2012). Carbon nanotubes in cancer therapy and drug delivery. J. Drug Deliv..

[55] Matea C.T., Mocan T., Tabaran F., Pop T., Mosteanu O., Puia C., Iancu C., Mocan L. (2017). Quantum dots in imaging, drug delivery and sensor applications. Int. J. Nanomed..

[56] Amerigos Daddy J.C.K., Chen M., Raza F., Xiao Y., Su Z., Ping Q. (2020). Co-Encapsulation of Mitoxantrone and β-Elemene in Solid Lipid Nanoparticles to Overcome Multidrug Resistance in Leukemia. Pharmaceutics.

[57] Billingsley M.M., Singh N., Ravikumar P., Zhang R., June C.H., Mitchell M.J. (2020). Ionizable Lipid Nanoparticle-Mediated mRNA Delivery for Human CAR T Cell Engineering. Nano Lett..

[58] Al Faruque H., Choi E.S., Lee H.R., Kim J.H., Park S., Kim E. (2020). Targeted removal of leukemia cells from the circulating system by whole-body magnetic hyperthermia in mice. Nanoscale.

[59] Mauz-Körholz C., Ströter N., Baumann J., Botzen A., Körholz K., Körholz D. (2018). Pharmacotherapeutic Management of Pediatric Lymphoma. Paediatr. Drugs.

[60] Zeng Z., Tung C.H., Zu Y. (2020). Aptamer-Equipped Protamine Nanomedicine for Precision Lymphoma Therapy. Cancers.

[61] Au K.M., Wang A.Z., Park S.I. (2020). Pretargeted delivery of PI3K/mTOR small-molecule inhibitor-loaded nanoparticles for treatment of non-Hodgkin’s lymphoma. Sci. Adv..

[62] Knapp C.M., He J., Lister J., Whitehead K.A. (2018). Lipid nanoparticle siRNA cocktails for the treatment of mantle cell lymphoma. Bioeng. Transl. Med..

[63] American Cancer Society Key Statistics for Osteosarcoma. https://www.cancer.org/cancer/osteosarcoma/about/key-statistics.html.

[64] American Cancer Society Osteosarcoma Early Detection, Diagnosis, and Staging. https://www.cancer.org/cancer/osteosarcoma/detection-diagnosis-staging/survival-rates.html.

[65] American Cancer Society Treating Osteosarcoma. https://www.cancer.org/cancer/osteosarcoma/treating.html.

[66] Abu Lila A.S., Ishida T. (2017). Liposomal Delivery Systems: Design Optimization and Current Applications. Biol. Pharm. Bull..

[67] Haghiralsadat F., Amoabediny G., Sheikhha M.H., Forouzanfar T., Helder M.N., Zandieh-Doulabi B. (2017). A Novel Approach on Drug Delivery: Investigation of A New Nano-Formulation of Liposomal Doxorubicin and Biological Evaluation of Entrapped Doxorubicin on Various Osteosarcoma Cell Lines. Cell J..

[68] Haghiralsadat F., Amoabediny G., Sheikhha M.H., Zandieh-Doulabi B., Naderinezhad S., Helder M.N., Forouzanfar T. (2017). New liposomal doxorubicin nanoformulation for osteosarcoma: Drug release kinetic study based on thermo and pH sensitivity. Chem. Biol. Drug Des..

[69] Skubitz K.M. (2003). Phase II trial of pegylated-liposomal doxorubicin (Doxil) in sarcoma. Cancer Investig..

[70] Caliskan Y., Dalgic A.D., Gerekci S., Gulec E.A., Tezcaner A., Ozen C., Keskin D. (2019). A new therapeutic combination for osteosarcoma: Gemcitabine and Clofazimine co-loaded liposomal formulation. Int. J. Pharm..

[71] Liu Q., Song Y., Duan X., Chang Y., Guo J. (2018). MiR-92a Inhibits the Progress of Osteosarcoma Cells and Increases the Cisplatin Sensitivity by Targeting Notch1. BioMed Res. Int..

[72] Huang W., Chen L., Kang L., Jin M., Sun P., Xin X., Gao Z., Bae Y.H. (2017). Nanomedicine-based combination anticancer therapy between nucleic acids and small-molecular drugs. Adv. Drug Deliv. Rev..

[73] Wang F., Pang J.D., Huang L.L., Wang R., Li D., Sun K., Wang L.T., Zhang L.M. (2018). Nanoscale polysaccharide derivative as an AEG-1 siRNA carrier for effective osteosarcoma therapy. Int. J. Nanomed..

[74] Haghiralsadat F., Amoabediny G., Naderinezhad S., Forouzanfar T., Helder M.N., Zandieh-Doulabi B. (2018). Preparation of PEGylated cationic nanoliposome-siRNA complexes for cancer therapy. Artif. Cells Nanomed. Biotechnol..

[75] Haghiralsadat F., Amoabediny G., Naderinezhad S., Zandieh-Doulabi B., Forouzanfar T., Helder M.N. (2018). Codelivery of doxorubicin and JIP1 siRNA with novel EphA2-targeted PEGylated cationic nanoliposomes to overcome osteosarcoma multidrug resistance. Int. J. Nanomed..

[76] Yin X., Chi Y., Guo C., Feng S., Liu J., Sun K., Wu Z. (2017). Chitooligosaccharides Modified Reduction-Sensitive Liposomes: Enhanced Cytoplasmic Drug Delivery and Osteosarcomas-Tumor Inhibition in Animal Models. Pharm. Res..

[77] Dirksen U., Brennan B., Le Deley M.C., Cozic N., van den Berg H., Bhadri V., Brichard B., Claude L., Craft A., Amler S. (2019). High-Dose Chemotherapy Compared with Standard Chemotherapy and Lung Radiation in Ewing Sarcoma with Pulmonary Metastases: Results of the European Ewing Tumour Working Initiative of National Groups, 99 Trial and EWING 2008. J. Clin. Oncol. Off. J. Am. Soc. Clin. Oncol..

[78] Le Deley M.C., Paulussen M., Lewis I., Brennan B., Ranft A., Whelan J., Le Teuff G., Michon J., Ladenstein R., Marec-Bérard P. (2014). Cyclophosphamide compared with ifosfamide in consolidation treatment of standard-risk Ewing sarcoma: Results of the randomized noninferiority Euro-EWING99-R1 trial. J. Clin. Oncol. Off. J. Am. Soc. Clin. Oncol..

[79] Paulussen M., Craft A.W., Lewis I., Hackshaw A., Douglas C., Dunst J., Schuck A., Winkelmann W., Köhler G., Poremba C. (2008). Results of the EICESS-92 Study: Two randomized trials of Ewing’s sarcoma treatment--cyclophosphamide compared with ifosfamide in standard-risk patients and assessment of benefit of etoposide added to standard treatment in high-risk patients. J. Clin. Oncol. Off. J. Am. Soc. Clin. Oncol..

[80] Kridis W.B., Toumi N., Chaari H., Khanfir A., Ayadi K., Keskes H., Boudawara T., Daoud J., Frikha M. (2017). A Review of Ewing Sarcoma Treatment: Is it Still a Subject of Debate?. Rev. Recent Clin. Trials.

[81] Meyers P.A. (2015). Systemic therapy for osteosarcoma and Ewing sarcoma. Am. Soc. Clin. Oncol. Educ. Book.

[82] Esiashvili N., Goodman M., Marcus R.B. (2008). Changes in incidence and survival of Ewing sarcoma patients over the past 3 decades: Surveillance Epidemiology and End Results data. J. Pediatr. Hematol. Oncol..

[83] PDQ Pediatric Treatment Editorial Board (2002). Ewing Sarcoma Treatment (PDQ^®^): Health Professional Version. PDQ Cancer Information Summaries.

[84] Baldwin P., Likhotvorik R., Baig N., Cropper J., Carlson R., Kurmasheva R., Sridhar S. (2019). Nanoformulation of Talazoparib Increases Maximum Tolerated Doses in Combination with Temozolomide for Treatment of Ewing Sarcoma. Front. Oncol..

[85] Smith M.A., Reynolds C.P., Kang M.H., Kolb E.A., Gorlick R., Carol H., Lock R.B., Keir S.T., Maris J.M., Billups C.A. (2015). Synergistic activity of PARP inhibition by talazoparib (BMN 673) with temozolomide in pediatric cancer models in the pediatric preclinical testing program. Clin. Cancer Res..

[86] Gu W., Wu C., Chen J., Xiao Y. (2013). Nanotechnology in the targeted drug delivery for bone diseases and bone regeneration. Int. J. Nanomed..

[87] Roelofs A.J., Thompson K., Gordon S., Rogers M.J. (2006). Molecular mechanisms of action of bisphosphonates: Current status. Clin. Cancer Res. Off. J. Am. Assoc. Cancer Res..

[88] Ramanlal Chaudhari K., Kumar A., Megraj Khandelwal V.K., Ukawala M., Manjappa A.S., Mishra A.K., Monkkonen J., Ramachandra Murthy R.S. (2012). Bone metastasis targeting: A novel approach to reach bone using Zoledronate anchored PLGA nanoparticle as carrier system loaded with Docetaxel. J. Control. Release Off. J. Control. Release Soc..

[89] Fontaine S.A.G., Houghton P., Kurmasheva R., Diolaiti M., Ashworth A., Peer C., Nguyen R., Figg W., Vera D.B., Santi D. (2020). A Very Long-Acting Poly(ADP-ribose)polymerase Inhibitor. Cancer Res..

[90] De Feo A., Sciandra M., Ferracin M., Felicetti F., Astolfi A., Pignochino Y., Picci P., Carè A., Scotlandi K. (2019). Exosomes from CD99-deprived Ewing sarcoma cells reverse tumor malignancy by inhibiting cell migration and promoting neural differentiation. Cell Death Dis..

[91] Zaritski A., Castillo-Ecija H., Kumarasamy M., Peled E., Sverdlov Arzi R., Carcaboso Á.M., Sosnik A. (2019). Selective Accumulation of Galactomannan Amphiphilic Nanomaterials in Pediatric Solid Tumor Xenografts Correlates with GLUT1 Gene Expression. ACS Appl. Mater. Interfaces.

[92] Alhaddad A., Adam M.P., Botsoa J., Dantelle G., Perruchas S., Gacoin T., Mansuy C., Lavielle S., Malvy C., Treussart F. (2011). Nanodiamond as a vector for siRNA delivery to Ewing sarcoma cells. Small.

[93] Bell J.B., Rink J.S., Eckerdt F., Clymer J., Goldman S., Thaxton C.S., Platanias L.C. (2018). HDL nanoparticles targeting sonic hedgehog subtype medulloblastoma. Sci. Rep..

[94] Iorio A.L., Ros M., Fantappiè O., Lucchesi M., Facchini L., Stival A., Becciani S., Guidi M., Favre C., Martino M. (2016). Blood-Brain Barrier and Breast Cancer Resistance Protein: A Limit to the Therapy of CNS Tumors and Neurodegenerative Diseases. Anti Cancer Agents Med. Chem..

[95] Tang W., Fan W., Lau J., Deng L., Shen Z., Chen X. (2019). Emerging blood-brain-barrier-crossing nanotechnology for brain cancer theranostics. Chem. Soc. Rev..

[96] Pardridge W.M. (2005). The blood-brain barrier: Bottleneck in brain drug development. NeuroRx.

[97] Wang S.S., Bandopadhayay P., Jenkins M.R. (2019). Towards Immunotherapy for Pediatric Brain Tumors. Trends Immunol..

[98] Leitner D.F., Connor J.R. (2012). Functional roles of transferrin in the brain. Biochim. Biophys. Acta.

[99] Jefferies W.A., Brandon M.R., Hunt S.V., Williams A.F., Gatter K.C., Mason D.Y. (1984). Transferrin receptor on endothelium of brain capillaries. Nature.

[100] Johnsen K.B., Burkhart A., Melander F., Kempen P.J., Vejlebo J.B., Siupka P., Nielsen M.S., Andresen T.L., Moos T. (2017). Targeting transferrin receptors at the blood-brain barrier improves the uptake of immunoliposomes and subsequent cargo transport into the brain parenchyma. Sci. Rep..

[101] Johnsen K.B., Burkhart A., Thomsen L.B., Andresen T.L., Moos T. (2019). Targeting the transferrin receptor for brain drug delivery. Prog. Neurobiol..

[102] Sonoda H., Morimoto H., Yoden E., Koshimura Y., Kinoshita M., Golovina G., Takagi H., Yamamoto R., Minami K., Mizoguchi A. (2018). A Blood-Brain-Barrier-Penetrating Anti-human Transferrin Receptor Antibody Fusion Protein for Neuronopathic Mucopolysaccharidosis II. Mol. Ther..

[103] Li X., Yang Y., Zhao H., Zhu T., Yang Z., Xu H., Fu Y., Lin F., Pan X., Li L. (2020). Enhanced In Vivo Blood-Brain Barrier Penetration by Circular Tau-Transferrin Receptor Bifunctional Aptamer for Tauopathy Therapy. J. Am. Chem. Soc..

[104] Paterson J., Webster C.I. (2016). Exploiting transferrin receptor for delivering drugs across the blood-brain barrier. Drug Discov. Today Technol..

[105] Wang X., Zhao Y., Dong S., Lee R.J., Yang D., Zhang H., Teng L. (2019). Cell-Penetrating Peptide and Transferrin Co-Modified Liposomes for Targeted Therapy of Glioma. Molecules.

[106] Furnari F.B., Fenton T., Bachoo R.M., Mukasa A., Stommel J.M., Stegh A., Hahn W.C., Ligon K.L., Louis D.N., Brennan C. (2007). Malignant astrocytic glioma: Genetics, biology, and paths to treatment. Genes Dev..

[107] Watanabe K., Tachibana O., Sata K., Yonekawa Y., Kleihues P., Ohgaki H. (1996). Overexpression of the EGF receptor and p53 mutations are mutually exclusive in the evolution of primary and secondary glioblastomas. Brain Pathol..

[108] Bublil E.M., Yarden Y. (2007). The EGF receptor family: Spearheading a merger of signaling and therapeutics. Curr. Opin. Cell Biol..

[109] Westphal M., Maire C.L., Lamszus K. (2017). EGFR as a Target for Glioblastoma Treatment: An Unfulfilled Promise. CNS Drugs.

[110] Yang W., Barth R.F., Wu G., Huo T., Tjarks W., Ciesielski M., Fenstermaker R.A., Ross B.D., Wikstrand C.J., Riley K.J. (2009). Convection enhanced delivery of boronated EGF as a molecular targeting agent for neutron capture therapy of brain tumors. J. Neuro Oncol..

[111] Roth P., Preusser M., Weller M. (2016). Immunotherapy of Brain Cancer. Oncol. Res. Treat..

[112] Sampson J.H., Maus M.V., June C.H. (2017). Immunotherapy for Brain Tumors. J. Clin. Oncol. Off. J. Am. Soc. Clin. Oncol..

[113] Sampson J.H., Gunn M.D., Fecci P.E., Ashley D.M. (2020). Brain immunology and immunotherapy in brain tumours. Nat. Rev. Cancer.

[114] Foster J.B., Madsen P.J., Hegde M., Ahmed N., Cole K.A., Maris J.M., Resnick A.C., Storm P.B., Waanders A.J. (2019). Immunotherapy for pediatric brain tumors: Past and present. Neuro Oncol..

[115] Kimiz-Gebologlu I., Gulce-Iz S., Biray-Avci C. (2018). Monoclonal antibodies in cancer immunotherapy. Mol. Biol. Rep..

[116] Mi Y., Smith C.C., Yang F., Qi Y., Roche K.C., Serody J.S., Vincent B.G., Wang A.Z. (2018). A Dual Immunotherapy Nanoparticle Improves T-Cell Activation and Cancer Immunotherapy. Adv. Mater..

[117] Carter T., Mulholland P., Chester K. (2016). Antibody-targeted nanoparticles for cancer treatment. Immunotherapy.

[118] Alibakhshi A., Abarghooi Kahaki F., Ahangarzadeh S., Yaghoobi H., Yarian F., Arezumand R., Ranjbari J., Mokhtarzadeh A., de la Guardia M. (2017). Targeted cancer therapy through antibody fragments-decorated nanomedicines. J. Control. Release Off. J. Control. Release Soc..

[119] Gajjar A., Bowers D.C., Karajannis M.A., Leary S., Witt H., Gottardo N.G. (2015). Pediatric Brain Tumors: Innovative Genomic Information Is Transforming the Diagnostic and Clinical Landscape. J. Clin. Oncol. Off. J. Am. Soc. Clin. Oncol..

[120] Wu C., Xu Q., Chen X., Liu J. (2019). Delivery luteolin with folacin-modified nanoparticle for glioma therapy. Int. J. Nanomed..

[121] Fan K., Jia X., Zhou M., Wang K., Conde J., He J., Tian J., Yan X. (2018). Ferritin Nanocarrier Traverses the Blood Brain Barrier and Kills Glioma. ACS Nano.

[122] Choi J., Rui Y., Kim J., Gorelick N., Wilson D.R., Kozielski K., Mangraviti A., Sankey E., Brem H., Tyler B. (2020). Nonviral polymeric nanoparticles for gene therapy in pediatric CNS malignancies. Nanomed. Nanotechnol. Biol. Med..

[123] Kim J., Dey A., Malhotra A., Liu J., Ahn S.I., Sei Y.J., Kenney A.M., MacDonald T.J., Kim Y. (2020). Engineered biomimetic nanoparticle for dual targeting of the cancer stem-like cell population in sonic hedgehog medulloblastoma. Proc. Natl. Acad. Sci. USA.

[124] Kievit F.M., Stephen Z.R., Wang K., Dayringer C.J., Sham J.G., Ellenbogen R.G., Silber J.R., Zhang M. (2015). Nanoparticle mediated silencing of DNA repair sensitizes pediatric brain tumor cells to γ-irradiation. Mol. Oncol..

[125] St. Jude Children’s Research Hospital Retinoblastoma. https://www.stjude.org/disease/retinoblastoma.html#:~:text=Retinoblastoma%20is%20a%20rare%20cancer,families)%20or%20non%2Dhereditary.

[126] U.S. National Library of Medicine Retinoblastoma. https://medlineplus.gov/genetics/condition/retinoblastoma/#causes2020.

[127] Bhavsar D., Subramanian K., Sethuraman S., Krishnan U.M. (2016). Management of retinoblastoma: Opportunities and challenges. Drug Deliv..

[128] Khademi R., Razminia A. (2020). Selective nano-thermal therapy of human retinoblastoma in retinal laser surgery. Nanomed. Nanotechnol. Biol. Med..

[129] Moradi S., Mokhtari-Dizaji M., Ghassemi F., Sheibani S., Amoli F.A. (2020). The effect of ultrasound hyperthermia with gold nanoparticles on retinoblastoma Y79 cells. Gold Bull..

[130] Katchinskiy N., Godbout R., Hatef A., Elezzabi A. (2018). Anti-EpCAM Gold Nanorods and Femtosecond Laser Pulses for Targeted Lysis of Retinoblastoma. Adv. Ther..

[131] Li Z., Guo Z., Chu D., Feng H., Zhang J., Zhu L., Li J. (2020). Effectively suppressed angiogenesis-mediated retinoblastoma growth using celastrol nanomicelles. Drug Deliv..

[132] Tabatabaei S.N., Derbali R.M., Yang C., Superstein R., Hamel P., Chain J.L., Hardy P. (2019). Co-delivery of miR-181a and melphalan by lipid nanoparticles for treatment of seeded retinoblastoma. J. Control. Release.

[133] Cancer.Net Wilms Tumor-Childhood: Statistics. https://www.cancer.net/cancer-types/wilms-tumor-childhood/statistics#:~:text=In%20the%20United%20States%2C%20about,ages%20of%203%20and%204.

[134] Markovsky E., Vax E., Ben-Shushan D., Eldar-Boock A., Shukrun R., Yeini E., Barshack I., Caspi R., Harari-Steinberg O., Pode-Shakked N. (2017). Wilms Tumor NCAM-Expressing Cancer Stem Cells as Potential Therapeutic Target for Polymeric Nanomedicine. Mol. Cancer Ther..

[135] Sosnik A., Carcaboso A.M. (2014). Nanomedicines in the future of pediatric therapy. Adv. Drug Deliv. Rev..

